# Visualization balloon occlusion-assisted technique in the treatment of large or giant paraclinoid aneurysms: A study of 17 cases series

**DOI:** 10.3389/fneur.2023.1094066

**Published:** 2023-01-27

**Authors:** Tingbao Zhang, Yuankun Cai, Lesheng Wang, Liu Yang, Zhengwei Li, Wei Wei, Yu Feng, Zhongwei Xiong, Yichun Zou, Weiyu Sun, Wenyuan Zhao, Jincao Chen

**Affiliations:** ^1^Department of Neurosurgery, Zhongnan Hospital of Wuhan University, Wuhan, Hubei, China; ^2^Brain Research Center, Zhongnan Hospital of Wuhan University, Wuhan, Hubei, China

**Keywords:** paraclinoid aneurysms, visualization, balloon occlusion-assisted technique, microsurgical clipping, hybrid operation

## Abstract

**Objective:**

Although balloon-assisted techniques are valuable in aneurysm clipping, repeated angiography and fluoroscopy are required to understand the location and shape of the balloon. This study investigated the value of visualization balloon occlusion-assisted techniques in aneurysm hybridization procedures.

**Methods:**

We propose a visualization balloon technique that injects methylene blue into the balloon, allowing it to be well visualized under a microscope without repeated angiography. This study retrospects the medical records of 17 large or giant paraclinoid aneurysms treated by a visualization balloon occlusion-assisted technique in a hybrid operating room. Intraoperative surgical techniques, postoperative complications, and immediate and long-term angiographic findings are highlighted.

**Results:**

All 17 patients had safe and successful aneurysm clipping surgery with complete angiographic occlusion. Under the microscope, the balloon injected with methylene blue is visible through the arterial wall. The position and shape of the balloon can be monitored in real time without repeated angiography and fluoroscopic guidance. Two cases of intraoperative visualization balloon shift and slip into the aneurysm cavity were detected in time, and there were no cases of balloon misclipping or difficult removal. Of 17 patients, four patients (23.5%) experienced short-term complications, including pulmonary infection (11.8%), abducens nerve paralysis (5.9%), and thalamus hemorrhage (5.9%). The rate of vision recovery among patients with previous visual deficits was 70% (7 of 10 patients). The mean follow-up duration was 32.76 months. No aneurysms or neurological deficits recurred among all patients who completed the follow-up.

**Conclusion:**

Our study indicates that microsurgical clipping with the visualization balloon occlusion-assisted technique seems to be a safe and effective method for patients with large or giant paraclinoid aneurysms to reduce the surgical difficulty and simplify the operation process of microsurgical treatment alone.

## Introduction

Paraclinoid aneurysms can be defined as intracranial aneurysms that originate between the distal dural ring and the initial segment of the posterior communicating artery (1). Some patients with paraclinoid aneurysms are likely to present visual acuity and field deficits caused by the aneurysms' proximity to the ophthalmic artery and optic, oculomotor, trochlear, and abducens nerves (2–5). Traditionally, paraclinoid aneurysms are treated with surgical clipping. For large (range from 10 to 25 mm in diameter) and giant (≥25 mm in diameter) paraclinoid aneurysms with a broad neck, calcification, and intraluminal thrombus, direct surgical clipping is complex. It is associated with high mortality and disability rates. Modern advancements in endovascular treatment for these aneurysms have replaced a large portion of surgical clipping. As a novel endovascular treatment, the pipeline embolization device (PED) has been demonstrated to be effective for wide neck and giant aneurysms (6–8). Although the emerging array of advanced endovascular treatment materials has shown exceptional surgical outcomes, the mass effect cannot be reduced, especially in large and giant aneurysms (4, 9–11). Moreover, endovascular therapy is usually not indicated for some patients who cannot tolerate regular anticoagulation therapy, such as in combination with gastrointestinal dysfunction.

A hybrid operating room, known as the “one-stop” operating room, is fully equipped with medical imaging devices, such as high-quality angiographic equipment (12). It created a new procedure named hybrid surgery, which combines endovascular interventions and open surgery, which inherits the advantages of two treatments. Some studies reported that the surgical procedure in treating internal cerebral large or giant aneurysms had an excellent clinical outcome and a low number of complications. In previous studies, a balloon occlusion-assisted technique was used to block the proximal end of the aneurysm or straddle the aneurysm neck better to control the blood flow of the parent artery. As the tension of a giant paraclinoid aneurysm decreases, aneurysmal clips can be precisely placed with promising results (13–16). However, repeated angiography and fluoroscopy are required to understand the location and shape of the balloon during this type of hybrid operation. Meanwhile, there are also some clinical and technical difficulties, such as balloon rupture and displacement, slippage of the distal end of the balloon into the aneurysm cavity, and being mistaken.

**Figure 1:**
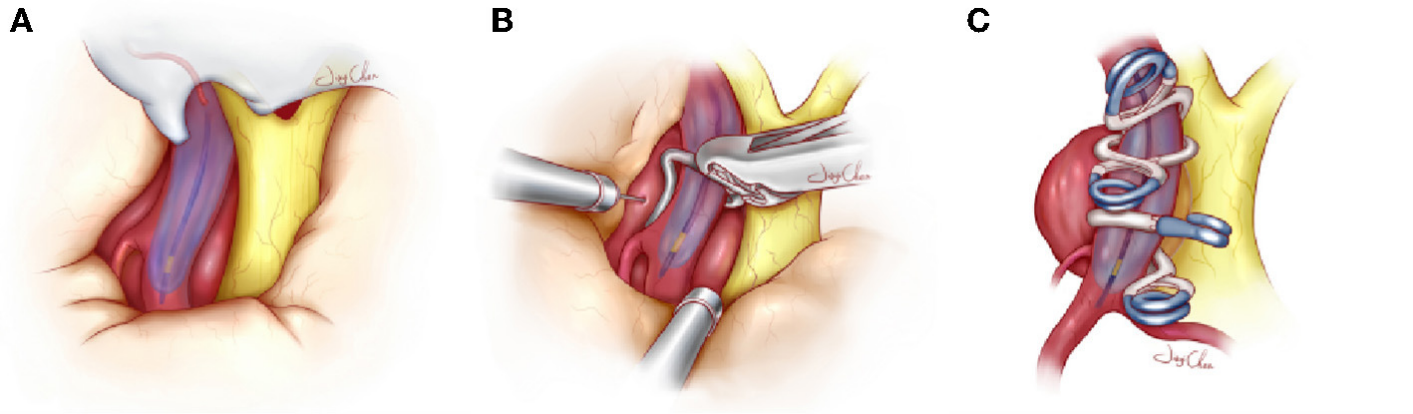
Schematic diagram about the visualization balloon occlusion-assisted technique used to treat large and giant paraclinoid aneurysms. **(A)** The balloon is successfully navigated across the neck of the aneurysm and inflated by a contrast agent diluted with methylene blue, which is identified under the microscope. **(B)** Once the balloon is inflated with the aid of microscopic visualization and the blood supply of the aneurysm has been completely blocked, the dome of the aneurysm is punctured or cut to release the remaining hematoma or thrombus. When the aneurysm collapses, the clots and plaques in the cavity are further removed, which facilitates accurate clip placement. **(C)** Accurate tandem clipping is performed with fenestrated clips or straight clips with the aid of the inflated balloon, which provides tactile and visual feedback to the surgeon. During clip closure, the surgeon can distinguish the vessel lumen and contiguous perforating branches from the longitudinal visualization balloon, and this helps to preserve the true lumen of the parent vessel and collateral blood vessels. Once the complete exclusion is confirmed, the deflated balloon and guide catheter are removed.

We propose a visualization balloon technique in which the balloon is injected with methylene blue, allowing it to visualize well under the microscope without repeated angiography (Figure 1). Moreover, since July 2018, we have been applying this technique in the hybridization operating room to treat paraclinoid aneurysm clipping. This study retrospects the medical records of 17 cases of visualization balloon-assisted technique for the treatment of large or giant paraclinoid aneurysms, reporting on the surgical technique and the results of the postoperative application of this technique.

## Material and methods

A retrospective analysis of 17 patients with a paraclinoid aneurysm treated with the visualization balloon occlusion-assisted technique in a hybrid operation room was performed between July 2018 and July 2020. The Wuhan University Zhongnan Hospital Research Ethics Committee approval was not required for this retrospective analysis of deidentified medical data. Written informed consent was waived by this Committee, as it was a retrospective analysis of our usual everyday work. The data of the patients were anonymized for this analysis. The confidential information of the patients was protected according to the national normative.

### Patient selection and data collection

Inclusion criteria were as follows: (1) Patients with large (10–25 mm in diameter) or giant (≥25 mm in diameter) aneurysms with a neck width of >5 mm and intraluminal thrombus, aneurysmal wall calcifications or atherosclerosis were included in the study. (2) The aneurysms were confirmed to be paraclinoid aneurysms by CT angiography (CTA) or digital subtraction angiography (DSA) before the operation. (3) No contraindications (heart failure, liver dysfunction, and renal dysfunction) for surgical intervention and craniotomy were present. (4) A one-stop operation involving balloon placement and aneurysm clipping was performed in the same hybrid operation room; no operations were performed in stages or multiple rooms. (5) Only patients in whom the balloon was placed at the neck of the aneurysm instead of the petrous internal carotid artery (ICA) or other positions were included in the study.

Seventeen patients were included in the final analyses. Patients' data were collected and statistically analyzed: basic information; aneurysm characteristics based on preoperative CTA or DSA; CT findings; intraoperative video data; intraoperative and postoperative DSA data; postoperative complications; follow-up data.

### Surgical procedures

**Figure 2:**
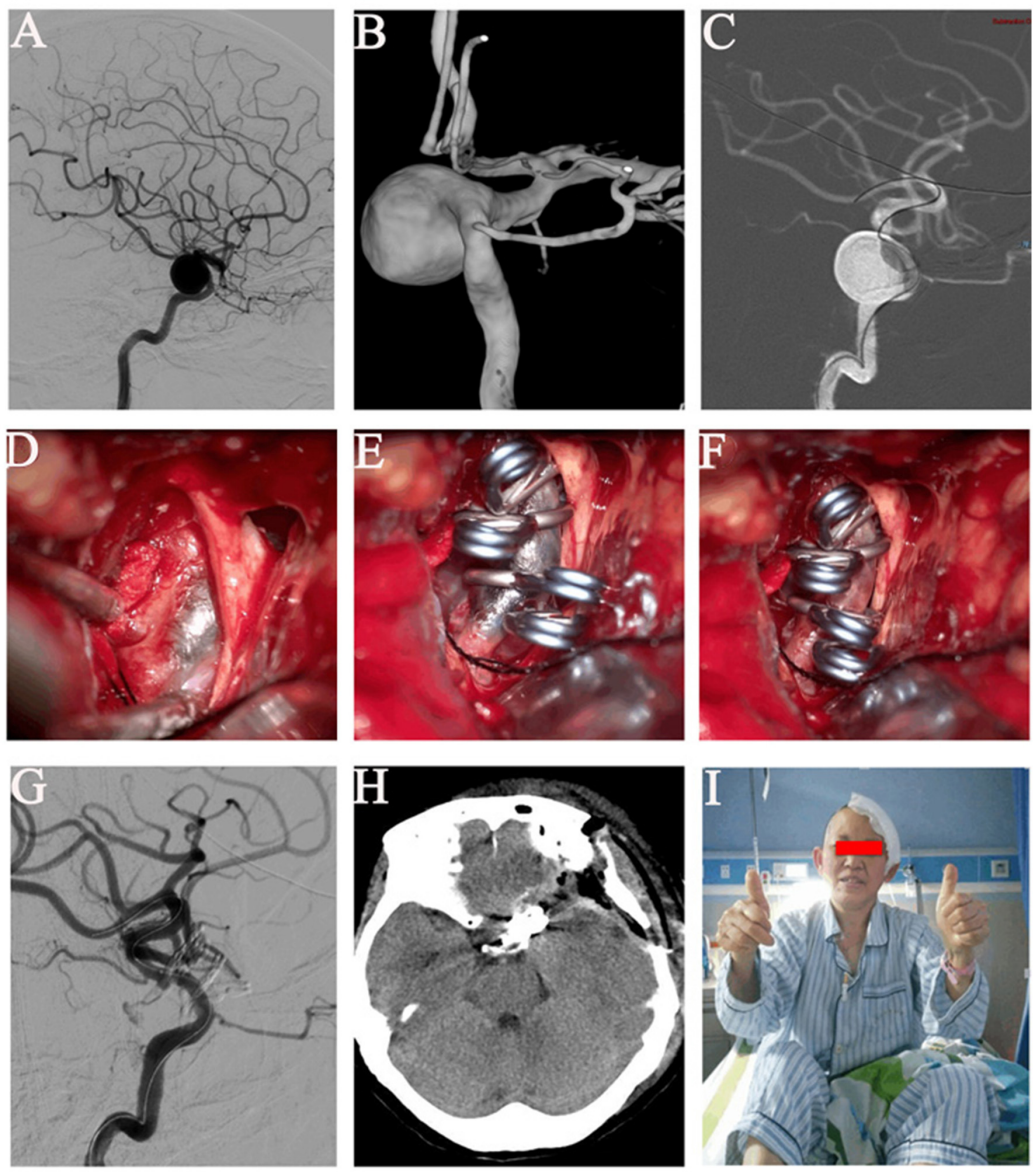
Images of a left giant paraclinoid aneurysm. **(A)** Angiography of a left wide-necked giant paraclinoid aneurysm of the internal carotid artery. **(B)** Three-dimensional digital subtraction angiography of the left wide-necked giant paraclinoid aneurysm. **(C)** The balloon was successfully inserted into the position of the neck of the aneurysm. **(D)** The balloon was dilated with methylene blue and could be clearly seen under the microscope, and the balloon adhered well to the wall. The blood supply of the aneurysm was completely blocked. **(E)** When the neck was blocked, the aneurysm was successfully clipped and the parent artery was reconstructed. **(F)** The balloon was withdrawn after the aneurysm had been satisfactorily clipped. **(G)** Digital subtraction angiography of the left internal carotid artery after successful clip occlusion showed no parent artery stenosis, no residual aneurysm, and a well-perfused distal vessel. **(H)** Head computed tomography on postoperative day 2. **(I)** This patient gained full consciousness without a neurological deficit.

The ICA balloon occlusion test (BOT), the cross circulation test, and Allcock's test was performed to understand cerebral blood flow compensation. Surgical procedures were performed using a biplane flat panel angiographic suite (UNIQ FD2020 Hybrid-OR, Philips, Eindhoven, The Netherlands) with 3D reconstruction under general anesthesia (Figures 2A, B). The surgical procedure was divided into the following steps:

(1) Balloon placement: After successfully placing the electrophysiological monitoring electrode and the catheter, the Seldinger technique was used to perform angiography of the lesion side carotid artery. Anteroposterior, lateral, and three-dimensional rotational views were obtained to clearly show the aneurysm's morphology, size, direction, and neck width. Next, a 4 × 20 mm Scepter C Occlusion Balloon (MicroVention, USA) was selected for placement at the aneurysm neck, ensuring that the balloon completely covered the aneurysm neck (Figure 2C). After placing the balloon, the contrast agent diluted with methylene blue (1 ml methylene blue was added to 10–20 ml of contrast agent) was used to expand the balloon tentatively, and the pressure required to block the blood supply of the aneurysm entirely was recorded. When satisfactory results were obtained, the balloon was deflated, and the guiding and balloon catheter was fixed correctly to prevent the balloon from shifting during the clipping process. To prevent intravascular thrombosis, the catheter was successively washed with heparinized saline during the clip occlusion procedure (500 ml normal saline:500 UI heparin).
(2) Microsurgical clipping of aneurysms with visualization balloon occlusion-assisted technique: After the balloon placement, standard craniotomy was performed with a pterion approach, and the sphenoid ridge and anterior clinoid process were resected in extradural. Generally, it was difficult to completely expose the body and neck of aneurysms with large volumes and high tension. The distal and proximal dural ring of the carotid artery and the outer membrane of the optic canal was sharply separated further to expand the space for exposure of the ICA. Exposure to essential ICA branches, such as the ophthalmic artery and posterior communicating artery, increased surgical safety. After the exposure, the interventional physician dilated the balloon with a contrast agent diluted with methylene blue at a suitable pressure (the experimentally recorded value at the time of balloon insertion before craniotomy). This allowed for clear visualization of the ICA under the microscope (Figure 2D). The blood flow to the aneurysm was blocked entirely (the blocking time was recorded). Usually no more than 5 min per attempt, and the obstruction time should be adjusted in real-time according to the changes observed by monitoring), and the tension of the aneurysm was decreased. If necessary, a puncture or minor incision was made for further decompression by suction. An appropriate aneurysmal clip was applied to reconstruct the ICA and clip the aneurysm while the important branches were well protected (Figure 2E).
(3) After successful clipping, the balloon was deflated and the blood flow to the parent artery was restored (Figure 2F). Angiography of the parent artery was performed after observation of aneurysm obliteration without bleeding. The angiography examination showed that the artery was well perfused with no residual aneurysm neck, no parent artery stenosis, and no inadvertently clipped branch vessel (Figure 2G). If any residual aneurysm, misclipping, or stenosis of the parent artery was present, adjustments were immediately made under the microscope until the angiography showed no problems. The balloon and guiding catheter were withdrawn after successful clip occlusion. After the operation, CT (Xper; Philips, Amsterdam, Netherlands) was immediately performed through the angiography equipment (Philips) to determine whether a hematoma or infarction was present (Figure 2H). This patient gained full consciousness without a neurological deficit (Figure 2I).

### Postoperative management and follow-up

After the operation, all patients returned to the neurosurgical intensive care unit (ICU) with endotracheal intubation and were placed on a ventilator to assist their breathing. With the blood pressure controlled, the perfusion maintained, the internal environment stabilized, moderate sedation and analgesia were performed, and the tracheal tube was removed.

Complete aneurysm occlusion was determined based on postoperative angiography. CT examination was performed the next day to determine whether hemorrhage or infarction was present. The sutures were removed on postoperative day 7, and the patients were discharged when they no longer had discomfort and had undergone all relevant examinations, including CTA. Regular follow-up was conducted after discharge, and DSA reexamination was generally performed 3–6 months after the surgery.

## Results

### Patient and aneurysm characteristics

Between July 2018 and July 2020, 17 patients with 17 large and giant paraclinoid aneurysms were treated using microsurgical clipping assisted by a visualization balloon occlusion-assisted technique (Table 1). A total of 6 men (35.3%) and 11 women (64.7%) with an average age of 59.65 years were enrolled in this study. A history of preoperative visual deficit, hypertension, diabetes, and coronary artery disease was present in 10 (58.8%), seven (41.2%), one (5.9%), and one (5.9%), respectively. Overall, there were four cases (23.5%) with subarachnoid hemorrhage (SAH).

**Table 1:** Summary of patient and aneurysm characteristics.

**No. of case**	**Age/Sex**	**Comorbidi-** **ties**	**Vision deficits**	**Aneurysm**					**Occlusion time (s)**	**Compli-** **cations**	**Follow-up (m)**	**mRS**	
				**Treatment history**	**SAH**	**Side**	**Diameter (mm)**	**Neck (mm)**				**Pre-operative**	**Post-operative**
1	65/F	No	Yes	No	Yes	R	21.6	10.7	424	Pulmonary infection	48	3	1
2	79/F	Hypertension, diabetes	No	No	No	R	17.5	8.5	415	No	44	1	0
3	58/F	No	Yes	No	No	L	23.6	11.4	367	No	41	0	0
4	47/M	No	No	Coiling	No	L	18.3	9.2	749	Pulmonary infection	40	1	1
5	58/M	Hypertension	No	No	No	L	14.5	7.1	302	No	39	0	0
6	55/M	Hypertension	Yes	No	No	R	26.7	11.7	558	Thalamus hemorrhage	38	1	2
7	53/F	No	No	No	No	R	19.7	9.4	289	No	35	1	0
8	63/M	Hypertension	Yes	No	No	L	13.3	6.9	267	No	32	0	0
9	50/F	Coronary heart disease	No	No	Yes	R	25.2	12.8	622	No	30	2	0
10	75/F	Hypertension	No	No	No	R	16.4	7.6	356	No	29	0	0
11	51/M	No	Yes	Coiling	Yes	L	22.8	10.4	737	Visual loss	28	4	1
12	75/F	No	No	Clipping	Yes	L	16.7	8.3	426	No	27	2	0
13	55/F	No	Yes	Coiling	No	L	23.3	10.6	683	No	26	1	0
14	48/M	No	Yes	No	No	L	27.4	11.4	655	No	26	1	0
15	73/F	Hypertension	Yes	No	Yes	L	14.8	7.4	297	VI paralysis	25	2	1
16	55/F	Hypertension	Yes	No	No	L	17.4	8.3	306	No	25	1	0
17	54/F	No	Yes	Coiling	No	L	22.1	9.6	591	No	24	1	0

There were 17 large or giant paraclinoid aneurysms treated with microsurgical clipping assisted by a visualization balloon occlusion-assisted technique. Eleven (64.7%) aneurysms were located on the left, and six (35.3%) were on the right. The aneurysm size ranged from 13.3 to 27.4 mm (mean, 20.08 mm). Fourteen (82.4%) aneurysms measured 10–25 mm, and three (17.6%) were ≥25 mm. The neck size of all aneurysms was >5 mm (mean, 9.49 ± 1.73 mm).

### Postoperative and follow-up

According to the postoperative angiography studies, all 17 lesions were excluded entirely from the parent vessel. All aneurysms were clipped (complete exclusion in 100% on follow-up angiography). There was no parent artery stenosis or distal embolization of an intraluminal thrombus.

The overall short-term disability rate was 23.5%. Two patients developed a pulmonary infection, and one exhibited abducens nerve paralysis. These complications were mild and were resolved entirely by pharmaceutical therapy after the operation. One patient presented with a small amount of thalamus hemorrhage, mixed aphasia, and contralateral limb hemiparesis after the operation. No severe complications occurred, such as postoperative carotid thrombosis or parent artery stenosis. Among the 17 patients, 10 (58.8%) had preoperative vision deficits. Seven patients recovered their vision after surgical decompression of the optic apparatus, and three patients had no improvement. The rate of vision recovery among patients with previous visual deficits was 70% (7 of 10 patients). Other than these instances, no new occurrence of visual impairment was observed in the other patients.

The follow-up duration ranged from 24 to 48 months (mean, 32.76 ± 7.57 months). Sixteen (94.1%) patients had good clinical outcomes [modified Rankin Scale (mRS) score of 0–1]. DSA examination showed no aneurysmal recurrence or new neurological deficits during the follow-up period.

## Discussion

For many years, treating aneurysms in the paraclinoid region has been a significant challenge for neurosurgeons because of the complex anatomical structure, narrow operative space, and high mortality and disability rates. In particular, large and giant aneurysms are often wide-necked and calcified and may contain an intraluminal thrombus. Direct clipping is not easy to be performed, and the incomplete aneurysm obliteration rate and parent artery stenosis rate are high, potentially reaching 15% (17). It is vital to control the proximal blood flow of the parent artery during the operation. Several studies have shown that the proximal blood flow of the parent artery can be controlled through cervical vascular exposure and blockage, which plays an essential promoting role in the surgical clipping of such aneurysms (14, 18, 19). However, because of the retrograde blood flow of the posterior communicating artery, ophthalmic artery, cavernous sinus segment of the ICA, and perforating branches, high tension of the aneurysm still exists. In this regard, some researchers have further introduced the balloon-assisted retrograde suction decompression method to reduce the aneurysm tension further (13, 15, 16, 20). This method makes surgical treatment of aneurysms in this segment possible. However, other reports have questioned its safety, proposing that this technology further increases the rate of embolic events, cerebral vasospasm, and other postoperative complications (21).

Since the beginning of the twenty-first century, with the development of hybrid operations and successful applications in neurosurgery, the advantages of neurological interventions and microsurgery have been effectively complementary. Neurosurgeons can not only perform microsurgeries but also directly conduct angiography, interventional embolization treatment, and immediate postoperative observation, which can significantly improve the success rate of surgery and reduce complications. For complex paraclinoid aneurysms, the balloon occlusion-assisted technique has been successfully performed by some neurosurgeons and neurointerventionists in hybrid operating rooms. In 2004, Thorell et al. (16) reported the successful clipping of giant paraclinoid aneurysms by the balloon occlusion-assisted technique at the level of the aneurysm neck, and no surgery-related complications were observed. In addition, the postoperative visual defects were improved. Other successful cases have since been reported in major clinical centers (15, 22). Chao et al. from West China Hospital of Sichuan University performed a clinical study in which 24 patients underwent microsurgical clipping with the balloon occlusion-assisted technique in the neck of the aneurysm, which achieved good clinical results (20). Nevertheless, repeated angiography and fluoroscopy are always needed during this hybrid operation. Meanwhile, these events, such as balloon rupture and displacement, slippage of the distal end of the balloon into the aneurysm cavity, and being mistaken, are inevitable.

In this study, we place the balloon on the level of the aneurysm neck in a way that covers the entire orifice of the aneurysm and thus wholly blocks the blood supply of the aneurysm, and then microclipping the aneurysm. This technique has two significant advantages. First, the balloon is placed by endovascular intervention, allowing it to quickly reach an area difficult to reach by craniotomy and effectively avoiding the trauma and pain caused by cervical vascular exposure. More importantly, the proximal and distal ends of the parent artery can be controlled more effectively simultaneously, blocking the parent artery's blood flow and nearby perforators. This can markedly reduce the tension of the aneurysm and provide more space for successful exposure of the aneurysm and adjacent perforator vessels. Even coils placed in the aneurysm, intraluminal thrombi, and sclerotic plaques can be removed to treat more complex aneurysms. This reduces bleeding and thrombotic events and thus decreases the incidence of complications. All aneurysms were safely clipped in our series. There were no cases of severe bleeding or distal embolization of an intraluminal thrombus.

Seventeen patients were treated with a Scepter balloon for the blocking in this study because it provided improved guidance in microsurgery after blocking, with better plasticity, more substantial flexibility, and more excellent controllability. After the inflation of the balloon, the aneurysm needed exposure under micromanipulation and the parent artery needed reshaping, which necessitated greater flexibility and plasticity of the balloon; these requirements were met by the Scepter balloon (MicroVention, USA). At the same time, methylene blue was added to the contrast agent filling balloon to allow the balloon in the internal carotid artery to be identified under the microscope. At least three aspects highlight the innovation of this technology. First, the position and adherence of the balloon can be observed in real time during the operation without intraoperative radiologic or fluoroscopic guidance. Second, through direct observation of the visualization balloon, the operator can avoid serious complications caused by accidental puncture and injury of the balloon in the process of puncture and aspiration of the tumor cavity or incision of the aneurysm for decompression. Third, the displacement and detaching of the balloon can be observed in real-time during the process of clip reconstruction, which can effectively avoid clipping mistakes in the balloon. In addition, the parent artery can be better reconstructed with the support of the filling balloon, and stenosis of the parent artery after surgery can be avoided effectively.

All 17 patients underwent methylene blue balloon dilatation and blocking during microsurgery in this series. No radiographic examination and repeated device switching were needed throughout the process, significantly simplifying the operative procedure. During the operation, two cases with balloon displacement and slippage into the aneurysm cavity were found in time and without balloon misclipping. All 17 aneurysms were successfully clipped, and angiography was performed immediately after the surgery. There was no residual aneurysm, parent artery stenosis, or perforator misclipping. The rate of good mRS score after the operation was 100%; at the same time, it also significantly improved the patients with preoperative visual impairment, with an improvement rate of 70%. Therefore, the visualization balloon occlusion-assisted technique is safe and effective for microsurgical clipping recurrent paraclinoid aneurysms after stent-assisted coil embolization.

## Limitations

First, there are inherent limitations in the present study since it is a retrospective study. Second, the number of cases in this study was small and there was only one observation group and no control group. Therefore, the study results need to be validated by case–control studies with strict inclusion and exclusion criteria.

## Conclusion

In consideration of patients with large or giant paraclinoid aneurysms, microsurgical clipping of complicated large and giant paraclinoid aneurysms with the visualization balloon occlusion-assisted technique in a hybrid operating room is a safe and effective combined surgical method. It significantly reduces surgical difficulty and simplifies the operation process. This technique can allow for accurate placement of aneurysmal clips, safely reconstruct the parent artery, relieve clinical symptoms, reduce complications, ease the mass effect, and reduce the high recurrence rate after embolization. Future clinical studies are needed to explore this finding further.

## Data availability statement

The original contributions presented in the study are included in the article/supplementary material, further inquiries can be directed to the corresponding authors.

## Ethics statement

The studies involving human participants were reviewed and approved by the Ethics Committee of Wuhan University. The patients/participants provided their written informed consent to participate in this study.

## Author contributions

JC and WZ: conceptualization and validation. JC: methodology, funding acquisition, and supervision. WS and YZ: software. YZ: formal analysis. LY: data curation. TZ and YC: writing the original draft preparation. YC and WW: writing, reviewing, and editing. ZL: visualization. ZX: project administration. All authors have read and agreed to the published version of the manuscript.
